# Boosting of Markers of Fcγ Receptor Function in Anti-HIV Antibodies During Structured Treatment Interruption

**DOI:** 10.1089/aid.2019.0047

**Published:** 2019-08-30

**Authors:** Hugh Billings, Bruce D. Wines, Wayne B. Dyer, Robert J. Center, Halina M. Trist, Stephen J. Kent, P. Mark Hogarth

**Affiliations:** ^1^ Immune Therapies Group, Life Sciences, Burnet Institute, Melbourne, Australia.; ^2^ Department of Immunology, Monash University Central Clinical School, Melbourne, Australia.; ^3^ Department of Pathology, The University of Melbourne, Melbourne, Australia.; ^4^ Australian Red Cross Blood Service, Alexandria, Australia.; ^5^ School of Medical Science, Faculty of Medicine and Health, University of Sydney, Sydney, Australia.; ^6^ Department of Microbiology and Immunology, Peter Doherty Institute for Infection and Immunity, University of Melbourne, Melbourne, Australia.; ^7^ Disease Elimination, Life Sciences, Burnet Institute, Melbourne, Australia.; ^8^ Department of Infectious Diseases, Melbourne Sexual Health Centre, Alfred Health, Central Clinical School, Monash University, Melbourne, Australia.

**Keywords:** Fc receptor, anti-envelope antibody, ART

## Abstract

Anti-HIV envelope (Env) antibodies elicit important Fc receptor functions, including FcγRIIIa-mediated natural killer cell killing of opsonized infected targets. How these antibodies evolve during HIV infection and treatment remains poorly understood. We describe changes in anti-HIV Env IgG using longitudinal samples from seroconverter subjects treated soon after infection and later during periods of structured treatment interruption (STI). Our well-validated dimeric rsFcγR binding assays combine effects of opsonizing antibody subclasses, epitopes, and geometries to provide a measure of FcγR (Fcγ receptor)-mediated functionality. IgG1 anti-Env titers diminished rapidly during antiretroviral therapy (ART; t_1/2_ 3.0 ± 0.8 months), while the dimeric rsFcγRIIIa activity persisted longer (t_1/2_ 33 ± 11 months), suggesting that there is maintenance of functional antibody specificities within the diminished pool of anti-HIV Env Abs. The initial antibody response to infection in two subjects was characterized by approximately fivefold higher FcγRIIIa compared with FcγRIIa binding activity. Uncoupling of FcγRIIa and FcγRIIIa activities may be a distinct feature of the early antibody response that preferentially engages FcγRIIIa-mediated effector functions. Two to three STI cycles, even with low viremia, were sufficient to boost dimeric FcγR activity in these seroconverter subjects. We hypothesize that increased humoral immunity induced by STI is a desirable functional outcome potentially achievable by therapeutic immunization during ART. We conclude that controlled viral antigen exposure under the protection of suppressive ART may be effective in eliciting FcγR-dependent function in support of viral reactivation and kill strategies.

## Introduction

Leukocyte activation through IgG binding to Fcγ receptors (FcγRs) is key to IgG-induced protective inflammatory responses, antibody-dependent cellular cytotoxicity (ADCC), antibody-dependent cellular phagocytosis (ADCP), antibody-dependent cellular viral inhibition, and antigen presentation.^1–4^ FcγR-mediated functions augment protective antibody responses to HIV infection in macaques, mice, and humans.^5,6^ A component of protection and viremic control in macaque vaccination and challenge studies is contributed by Fc-dependent functions, such as ADCC and ADCP.^7–11^ In addition to neutralizing activity, BnAbs clear infected cells mediated through FcγR binding^12^ and stimulation of the endogenous antibody response.^13,14^ However, highly protective BnAbs are rarely found in long-term infected individuals and are not induced by current immunization strategies. Hence, FcγR-mediated functions may be of particular importance early in infections.

Only one human vaccine trial (RV144) demonstrated efficacy against HIV-1 infection and this was mediated by antibody FcγR binding. Protective efficacy was a low 31.2% and short-lived,^15^ and ADCC/Fc effector functional antibodies in the absence of an IgA response correlated with protection,^15–17^ whereas neutralizing antibody and cytotoxic T lymphocyte (CTL) responses were comparatively weak.^18^ In contrast to the unsuccessful VAX003 trial, the RV144 vaccine generated non-neutralizing Abs with multiple FcγR functions, with higher IgG1 and IgG3 levels.^19^ These studies support a critical role for IgG FcγR binding activity in protection from HIV infection.

Combination antiretroviral therapy (ART) has effectively controlled HIV replication and limited transmission for over two decades,^20^ but a cure remains elusive. Potential cure strategies based on shock and kill approaches are in early stages of investigation.^21^ Complete clearance of the viral reservoir may depend on an immune component^22,23^ and is likely to require Fc-mediated effector functions for optimal efficacy.^24,25^

ART commenced early in HIV-1 infection reduces anti-HIV IgG antibody-secreting cells^26^ and serum antibodies,^27,28^ which is opposite to what may be required for virus reactivation and cure strategies. Moreover, the impact of ART on the half-life of anti-Env Ab titers and ADCC functional antibodies is poorly defined.^29,30^ Since Ab titers rebound more rapidly during ART interruption compared with initial infection,^28^ and HIV+ individuals on ART respond well to influenza A vaccination,^31^ vaccination to boost antibodies mediating ADCC could form part of an eradication strategy in patients on suppressive ART.

We investigated engineered dimeric ectodomains of FcγRs as functional markers of the humoral response to HIV. These bind closely spaced IgG Fc pairs to mimic the engagement and cross-linking of FcγR pairs by IgG-opsonized virus or infected cells.^32–36^ Binding is further influenced by the IgG subclass and, in the case of FcγRIIIa, glycosylation of the Fc domain.^37^ We examined longitudinal serum samples from HIV-infected subjects, who commenced ART close to the time of seroconversion, to address the Fc receptor-mediated functionality of anti-HIV Ab responses early in infection, their decline during ART, and boosting during structured treatment interruption (STI). The combined effects of host immune responses on viral containment are discussed in the context of viral eradication strategies.

## Materials and Methods

### Study participants

We recruited newly infected individuals to a study of antiviral immunity during ART. Baseline specimens were obtained before or shortly after commencing ART. Serum and plasma specimens were provided by the Immunovirology Research Network of the Australian Centre for HIV and Hepatitis Virology Research and the Victorian HIV Blood and Tissue Storage Bank, Alfred Hospital. The respective human research ethics committees approved the study. Three patients subsequently participated in a pilot assessment of STI aimed at boosting antiviral immunity, after at least 6 months of undetectable viremia on ART. The number of patients given STI was small because we discontinued recruitment for this pilot after reports that time-based STI could result in viral reservoir reseeding and induce viral resistance.^38^ Patients on continuous ART and patients with poor treatment adherence (e.g., SC49) acted as comparators for STI participants. Blood specimens were taken on monthly visits or weekly during the STI phase.

### Reagents, Abs, and HIV-1 antigens

Albumin, bovine fraction V (BSA), and casein sodium salt were from Sigma-Aldrich. A chimeric IgG1 comprising a mouse leader and VH sequence (from TIB142 antitrinitrophenyl [TNP]; American Type Culture Collection) joined to a human IgG1 C region sequence (IgG1 anti-TNP) was previously described.^39^ Biotin mouse anti-huIgG1 (A10650, clone HP6069), biotin mouse anti-huIgG3 (05-3640, clone HP6047), and 3,3′,5,5′-tetramethylbenzidine (TMB) enzyme-linked immunosorbent assay (ELISA) substrate and the high-sensitivity streptavidin–horseradish peroxidase (HRP) conjugate were from Thermo Fisher (Melbourne, Australia). HIV-1 gp140 AD8 was as previously described.^40,41^

### Determination of rsFcγR binding by Ab-opsonized gp140 in the ELISA

ELISA plates (MaxiSorp; Nunc) were coated with HIV-1 Env antigen gp140-AD8 Env or gp120^40,41^ in phosphate-buffered saline (PBS)/1 mM EDTA (PBSE) at 50 ng/50 μL per well and blocked with PBSE/5% casein sodium salt (Sigma-Aldrich). Patient samples were incubated with 1% NP40 at 42°C for 30 min, appropriately diluted in PBSE/1% BSA, and incubated in wells for 2 h at 37°C. MAbs b12, PGT121, and 2G12 were kindly provided by Dennis Burton, The Scripps. Plates were then incubated with biotinylated rsFcγRIIa (H131 allele at 0.2 μg/mL) or rsFcγRIIIa (V158 allele at 0.1 μg/mL) and detected with TMB, as described previously.^37^

### Determination of IgG subclass levels among Ab-opsonized gp140 titers

Anti-gp140-AD8 titers were measured in NP40-treated serum/plasma, diluted 1:20 to 1:6,400 for IgG1. IgG3 was not detected at high dilution as was IgG1, and IgG3 levels are reported as absorbance units (AU) at 1:5 dilution. Detection was performed with biotinylated mouse anti-huIgG1 (A10650, clone HP6069; Thermo Fisher) or mouse anti-IgG3 (05-3640, clone HP6047; Thermo Fisher) at 1 μg/mL in PBS/1% BSA for 1 h at 37°C, followed by 1:10,000 high-sensitivity streptavidin-HRP conjugate (Pierce, Thermo Fisher) in PBS/1% BSA for 1 h, then developed with TMB.

### Antiviral T cell responses

Assays for CD4 T cell proliferative responses to HIV-1 p24 antigen and CD8 responses determined by IFN-γ ELISPOT are described elsewhere.^42,43^

### Data and statistical analysis

**FIG. 1.**
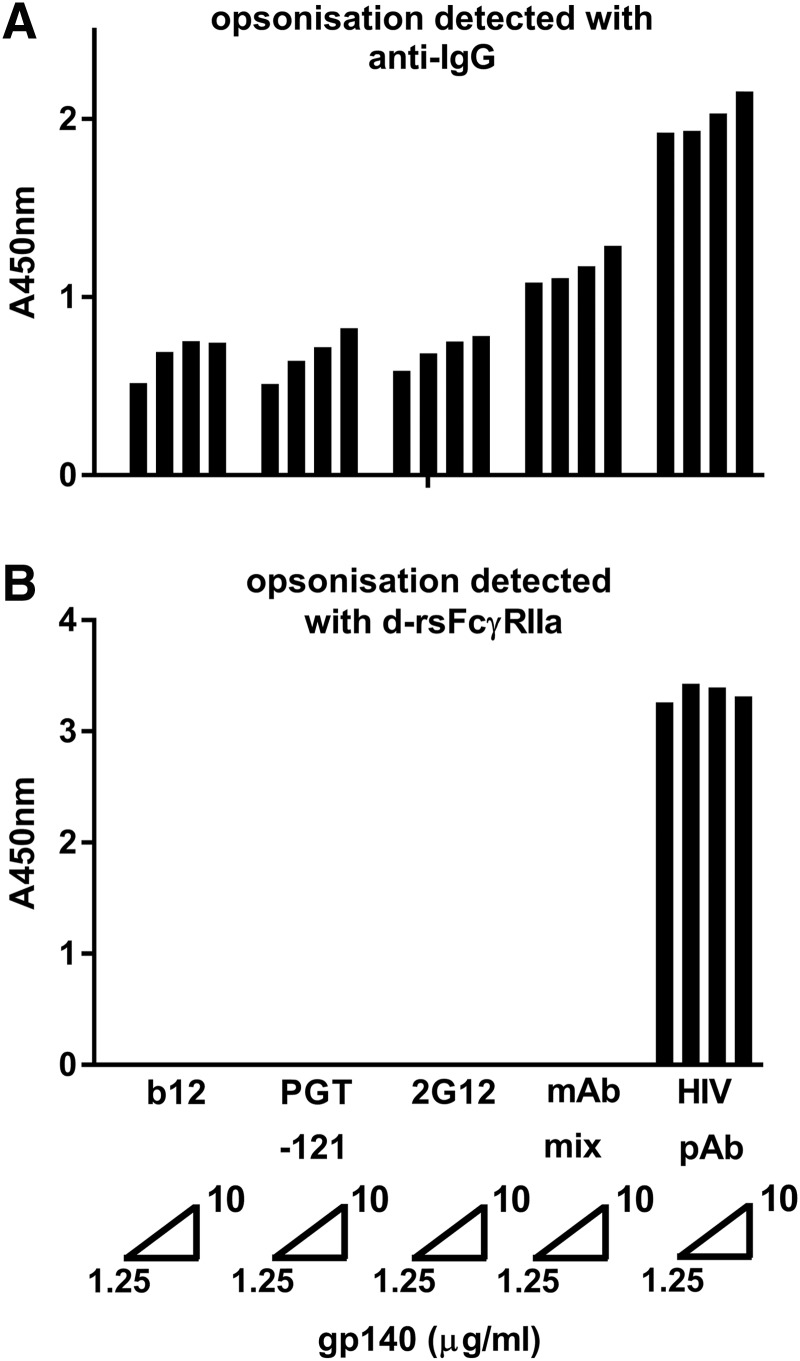
rsFcγRIIa dimer binding to IgG-opsonized envelope is dependent on the clonal diversity (polyclonality) of opsonizing Abs. HIV Env gp140-AD8 was coated at concentrations of 1.25, 2.5, 5, or 10 μg/mL and opsonized with HIV+ patient serum at 1/2,000 dilution (∼5–10 μg/mL total Abs) or three mAb IgG antibodies, separately (at 20 μg/mL) and in combination (mAb mix, 60 μg/mL total), **(A)** anti-IgG binding and **(B)** dimeric rsFcγRIIa-H131 binding.

Statistical analysis and curve fitting (Table 1) were performed with GraphPad Prism, version 7 (GraphPad, San Diego, CA). Dimeric rsFcγR binding activities are reported as AU background (Figs. 1 and 4-FcγRIIa only) or as AU normalized to rsFcγR binding to IgG1 anti-TNP^37^ at 0.5 μg/mL. Differences in binding activity were determined by Mann–Whitney *t*-tests (Fig. 3) or ANOVA.

**Table 1.** Baseline Characteristics and Decline in Serological Parameters During Antiretroviral Therapy

*Subject*	*CD4 baseline*^a^	*CD4, average ± SD*	*Initial VL*^b^	*Initial IgG1 titer DF50*^c^	*Half-life of serological parameters, t_1/2_ (months)*			
					*IgG1 titer*^d^	*Dimeric rsFcγR binding*^e^		
						*FcγRIIa*	*FcγRIIb*	*FcγRIIIa*
SC22	800	903 ± 193	3,800	6,400	3.9 (0.7)^f^	8.1 (0.6)	9.4 (0.9)	54 (0.5)
SC61	510	704 ± 152	6,200	6,000	3.8 (0.7)	13.3 (0.5)	22.1 (0.9)	70 (0.6)
SC68	800	799 ± 222	460,000	3,200	0.5 (0.6)	3.4 (0.8)	3.2 (0.9)	4.5 (0.9)
SC15	610	935 ± 189	190,000	2,200	3.3 (0.9)	12.5 (0.6)	4.2 (0.9)	15.5 (0.8)
SC24	471	582 ± 126	UD	1,200	0.7 (0.9)	42.6 (0.7)	ND	85.7 (0.7)
SC74	670	831 ± 201	UD	820	1.7 (0.6)	6.6 (0.9)^f^	1.5 (0.9)	16 (0.8)
SC21	736	928 ± 229	UD	473	7.7 (0.9)	4.2 (0.8)	ND	12.4 (0.8)
SC84	722	860 ± 150	UD	188	ND	ND	ND	ND
SC49	430	764 ± 178	3,840	97	2.6 (0.9)^f^	ND	ND	1.7 (0.9)^f^
Mean ± SEM	639 ± 47	812 ± 39		2,290 ± 811	3.0 ± 0.80	13.0 ± 5.0	8.1 ± 3.7	33 ± 11

Values in brackets are *r*^2^ correlation coefficients for curve fitting.

^a^ CD4 counts for SC24 and SC21 were determined 5 and 0.4 months after study commencement, averaged counts are across the whole study period.

^b^ VL, viral load at the start of the study. Viremia was UD in four subjects who commenced ART before baseline assessment.

^c^ IgG1 titer is defined as the dilution factor giving 50% of maximal signal; half-lives of serological parameters during ART were determined by curve fitting.

^d^ To agonist versus response curve (variable slope).

**FIG. 2.**
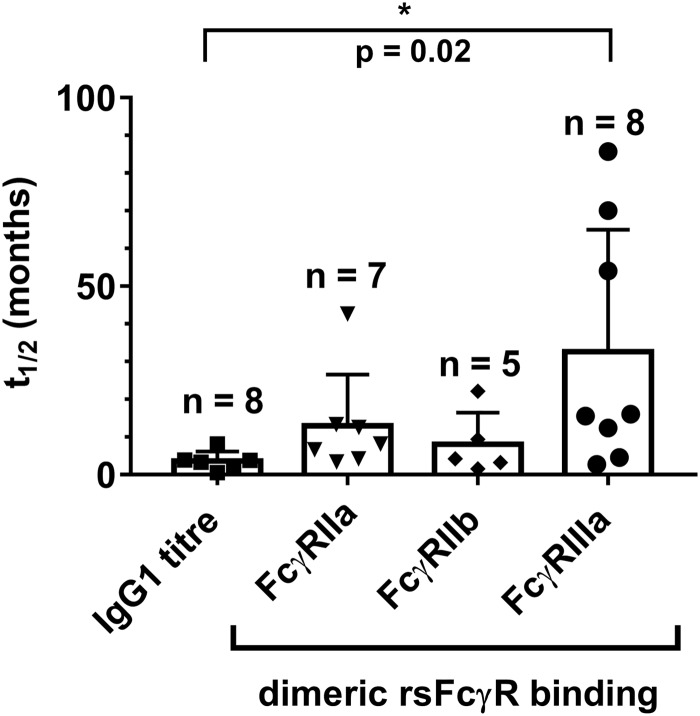
Anti-Env IgG1 titer and dimeric rsFcγR binding activity decline during ART. Mean half-lives (t_1/2_) ± SEM (*n* = 5–8 as indicated), one-way ANOVA, Tukey's multiple comparison test, *p < .05. ART, antiretroviral therapy; SEM, standard error of the mean.

^e^ To inhibitor versus response curves (also Figs. 1 and 2).

^f^ Determined by linear fit.

ART, antiretroviral therapy; ND, not determined; SD, standard deviation; SEM, standard error of the mean; UD, undetectable.

## Results

### The dimeric rsFcγR assay and anti-IgG are different measures of gp140 opsonization

Saturating levels of monoclonal (20 μg/mL) or polyclonal antibodies (pAbs) were used to opsonize AD8 gp140. Binding of the IgG1 mAbs and pAbs to gp140 was detected with anti-IgG (Fig. 1A), and the capacity of this opsonized gp140 to bind dimeric rsFcγRIIa was also determined (Fig. 1B). While the level of bound IgG was lower for mAbs than pAbs, only the pAb-opsonized gp140 bound dimeric rsFcγR. In contrast, the bound mAbs, separately or in combination, were undetected by the dimeric rsFcγR reagent. Thus, in subsequent dimeric rsFcγR assays, low dilution of samples was used to allow high occupancy of gp140 and so favor formation of pairs of opsonizing IgG antibodies that support dimeric receptor binding.

### Decline in humoral responses in seroconverters on ART

IgG1 is the predominate antibody binding FcγRs and so the effect of early ART on anti-HIV Env IgG1 titers and dimeric rsFcγR binding activity was determined (Table 1). The initial IgG1 anti-Env titers were very low in subjects who were either treated very early (e.g., SC49) or treated before the baseline sample was taken (e.g., SC74). Uninterrupted ART controlled the viral load (VL) to undetectable levels in the majority of subjects as expected, and uninterrupted ART was associated with a decline of the IgG1 anti-Env titer (DF_50_ of 2,550 ± 870) with a short half-life (t_1/2_) of 3.0 ± 0.8 months (Table 1). At low dilution, the loss of Env-specific pAb IgG capable of FcγRIIIa dimer binding was slower (*p* = .023) with a t_1/2_ of 33 ± 11.0 months. This indicated that the diversity of IgG antibody specificities comprising the pAb response, necessary for dimeric rsFcγR binding, was relatively retained during the decline of antibody titer (Table 1 and Fig. 2). The loss of dimeric FcγRIIa binding activity also trended toward a slower decline with t_1/2_ of 13.0 ± 5.0 and 8.1 ± 3.7 months for dimeric rsFcγRIIa and dimeric rsFcγRIIb, respectively.

**FIG. 3.**
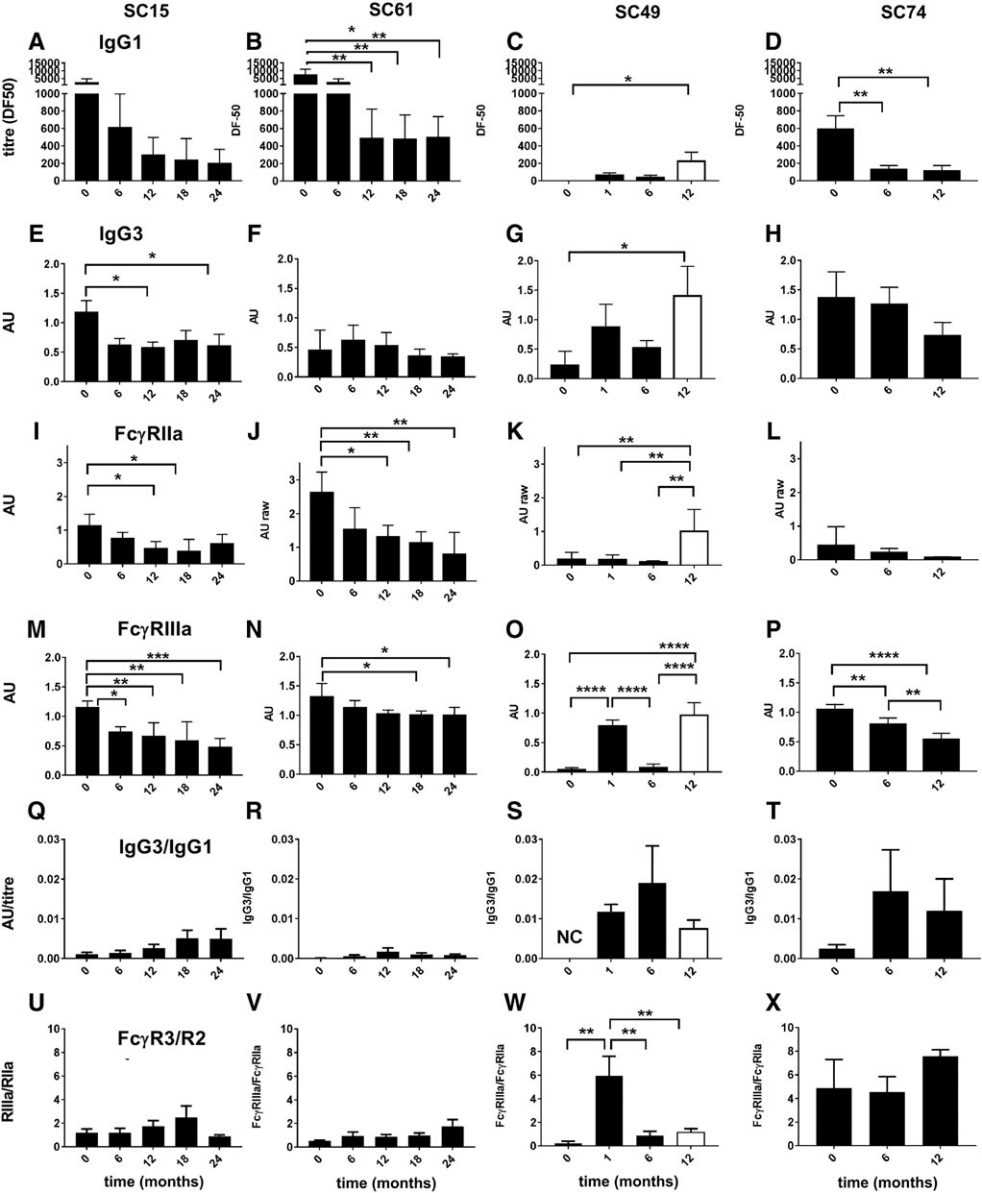
The anti-HIV IgG response and FcγR activities were detected in SC samples by ELISA using immobilized AD8-gp140; **(A–D)** opsonizing IgG1 titer, mean DF50 ± range (*n* = 3); **(E–H)** opsonizing IgG3, AU ± range (at 1/5 dilution, *n* = 3); **(I–P)** dimeric rsFcγR binding to Env opsonized with serum samples or plasma at 1/50 dilution was measured and **(I–L)** dimeric rsFcγRIIa activity presented as mean AU ± SEM, *n* = 4; **(M–P)** dimeric rsFcγRIIIa activity (mean normalized AU ± SEM, *n* = 4); **(Q–T)** the ratio of IgG3, AU/IgG1 titer, mean DF50; and **(U–X)** the ratio of dimeric rsFcγRIIIa activity/dimeric rsFcγRIIa activity. The *open bars* indicate an off-ART sample for SC49. Change from baseline was assessed by one-way ANOVA, Tukey's multiple comparison test, *p < .05, **p < .01, ***p < .001, and ****p < .0001. AU, absorbance units; ELISA, enzyme-linked immunosorbent assay; FcγR, Fcγ receptor; SC, seroconverter.

Features of the humoral response were contrasted for two subjects, SC15 and SC61, with stronger initial anti-Env IgG1 titers (DF50 2,200 and 6,000, respectively) and two subjects, SC49 and SC74, with undetectable VL at baseline and low initial IgG1 responses (DF50 97 and 820, respectively). In SC49 and SC74, the early FcγRIIIa activity was dominant (Fig. 3O, P) and FcγRIIa activity (Fig. 3K, L) was low, with a ratio of FcγRIIIa/FcγRIIa binding of ∼6 for SC49 and SC74 (Fig. 3W, X). Furthermore, although IgG3 levels were low, the ratio of IgG3/IgG1 (AU/titer) was up to 10-fold higher in SC49 and SC74 (Fig. 3S, T) compared with SC15 and SC61 (Fig. 3Q, R).

### Humoral response in a subject with viral rebound during poor ART compliance

**FIG. 4.**
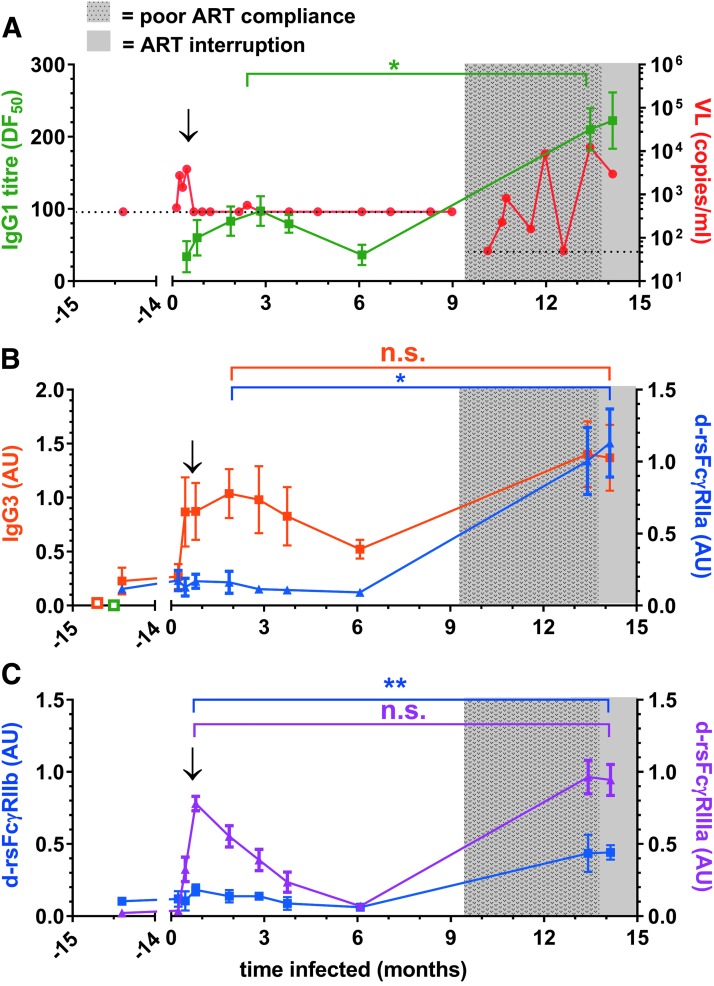
The initial anti-HIV IgG response of patient SC49 had high dimeric rsFcγRIIIa activity and low FcγRII binding activity. **(A)** *Left* axis (*green* symbols) AD8-gp140 opsonizing IgG1 titer, mean DF50 ± range (*n* = 3); *right* axis (*red* symbols) viral load (Roche) is shown with *dotted lines* for the assay detection limits at 400 and subsequently 50 viral copies per milliliter. **(B)** *Left* axis (*orange closed* symbols), opsonizing IgG3 levels at 1/5 dilution, mean AU ± range, *n* = 3, *open* symbols show HIV−human serum samples (*n* = 3), background anti-IgG1 (*green*), and anti-IgG3 (*orange*) binding to gp140; *right* axis, dimeric rsFcγRIIa-H131, AU binding to AD8-gp140 opsonized at 1/50 sample dilution, mean ± SEM, *n* = 4. **(C)** *Left* axis, dimeric rsFcγRIIb binding (*blue* symbols), normalized AU, and *right* axis *filled triangle* (*magenta*) dimeric rsFcγRIIIa-V158 binding, normalized AU. The *arrow* marks the time of ART initiation, and the period of poor ART compliance is shown by a hatched *gray* background pattern and complete ART removal is indicated by the *plain gray* background. Means for the early and late peak activities were compared using an unpaired Mann–Whitney *t*-test, *p < .05 and **p ≤ .01.

Suppressive ART very early after infection in SC49 controlled a brief viremia, with low anti-Env IgG1 titer and IgG3 levels peaking around 2–3 months (Fig. 4A, B). These humoral responses declined during ART, but were boosted upon viremia during a period of poor ART compliance (>9 months). Dimeric rsFcγRIIa-H131 binding activity was initially low (Figs. 3K and 4B), but increased 20-fold (*p* < .05) during poor ART compliance, while the IgG1 titer only increased twofold (Fig. 4A, B). Likewise, dimeric rsFcγRIIb activity was also weak during the initial phase (Fig. 4C), but increased fourfold during poor ART compliance (*p* < .01).

Unlike dimeric rsFcγRII activity, dimeric rsFcγRIIIa binding activity was robust in the early humoral response to infection (Figs. 3O and 4C), peaking around 1 month postinfection. During poor ART compliance, dimeric rsFcγRIIIa binding activity increased to only 1.3-fold higher than the initial peak activity (Fig. 4C). Thus, in the initial response to infection, dimeric rsFcγRIIIa activity was greater than that of dimeric rsFcγRII.

### Boosted humoral response in a subject that failed to control viremia during STI

**FIG. 5.**
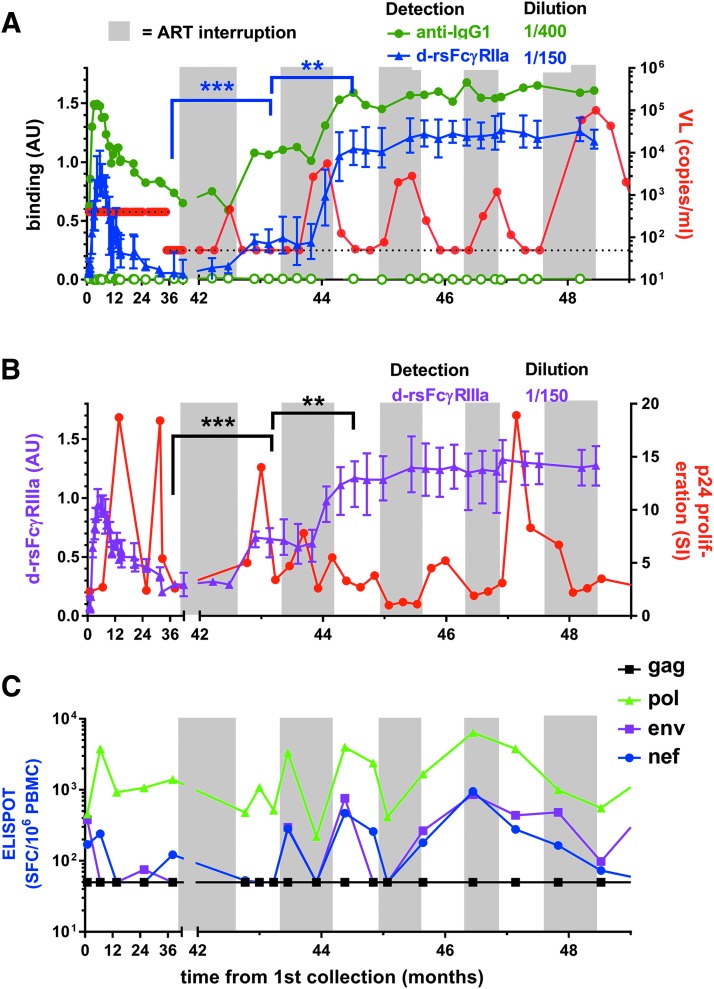
STI in subject SC21 allowed viral rebound and stimulated stepwise boosting of anti-Env IgG1 and dimeric rsFcγR activity. **(A)** *Left* axis, dimeric rsFcγRIIa-H131 binding to Env opsonized with a 1/150 dilution of samples (normalized AU, mean ± range, *n* = 3, *blue* symbols) and anti-IgG1 binding to a 1/400 dilution of samples with and without gp140 (*green closed* and *open* symbols, respectively). *Right* axis, viral load (*red* symbols) is shown with *dotted lines* indicating the assay detection limits at 400 and subsequently 50 viral copies per milliliter. Assay upper limit is 750,000 copies/mL. At recruitment, SC21 had been on ART for 1 month and periods of STIs are shown with a *gray* background. **(B)** *Left* axis, dimeric rsFcγRIIIa binding (normalized AU, magenta symbols, *n* = 3). *Right* axis, p24-stimulated T cell proliferation (*red* symbols) and **(C)** IFNγ ELISPOTs/10^6^ PBMCs stimulated with gag, pol, env, and nef peptides. Dimeric rsFcγR activities at the indicated times were compared using an unpaired Mann–Whitney *t*-test, **p ≤ .01 and ***p < .001. STI, structured treatment interruption.

Subject SC21 commenced ART 1 month before the study and had undetectable baseline VL. Viral rebound occurred during each STI, and extremely high viral rebound occurred during the final interruption cycle, prompting permanent resumption of ART (Fig. 5). Increased IgG1 levels and dimeric rsFcγRIIa and dimeric rsFcγRIIIa binding activities occurred within weeks of low viral recrudescence during the first ART interruption (VL = 460 copies/mL) and second ART interruption cycles (Fig. 5A, B).

The kinetics of HIV-specific T cell responses relative to FcγR-mediated Ab function during early treatment and ART interruption is not known. The p24 proliferative response (a marker of HIV-specific CD4 T cell responses) was sensitive to viral replication and declined after increased viremia (Fig. 5B), while the IFNγ ELISPOT response (generally a marker of CD8 T cell responses), particularly to Pol peptides, remained relatively robust (Fig. 5C). Thus, while a small number of STIs boosted the dimeric rsFcγR binding activity and increased T cell responses, these did not prevent the high viral rebound, which was only controlled by resumption of ART.

### Boosted immune responses in subjects with viral control

**FIG. 6.**
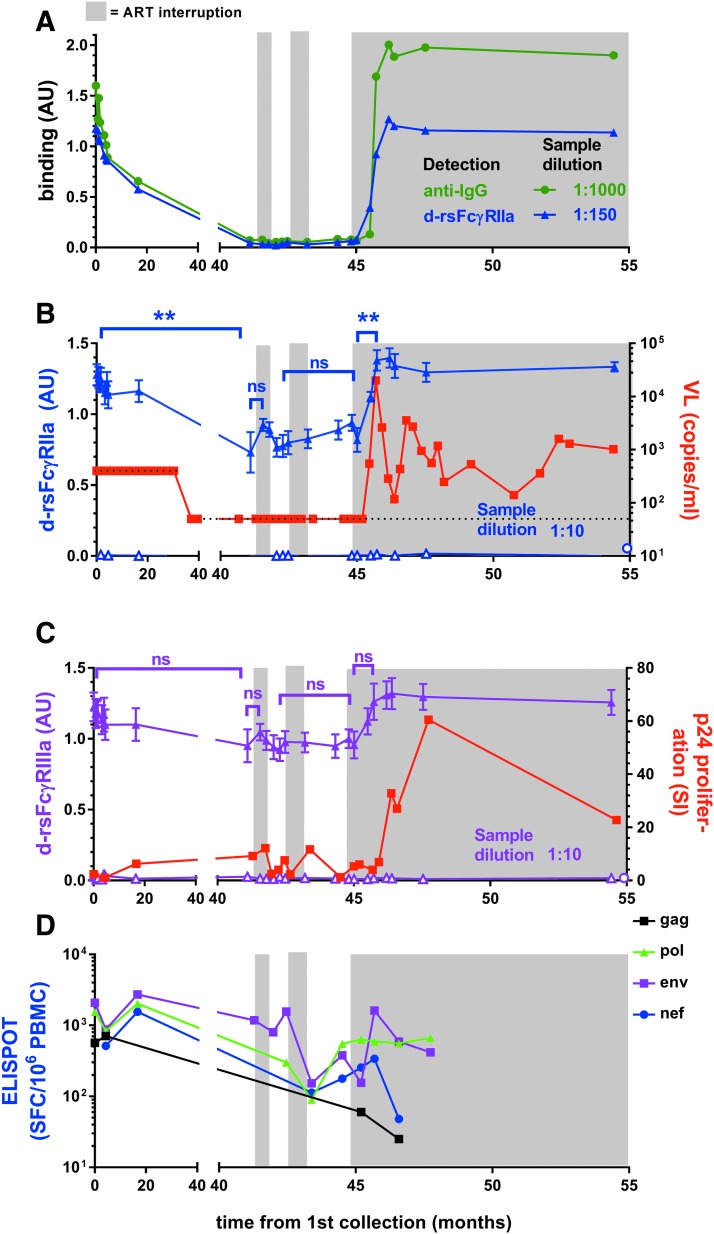
Boosted Ab, dimeric rsFcγR activity, and T cell immunity in STI in subject SC24 are contemporaneous with partial viral control. At recruitment, SC24 had been on ART for 6 months. STI periods of no ART are shown with the *gray* background. **(A)** Anti-IgG and dimeric rsFcγRIIa binding to Env opsonized at 1/1,000 or 1/150 sample dilution, respectively, is shown for 1 representative experiment. **(B, C)** *Left* axis, dimeric rsFcγR binding (filled *triangles*) to Env opsonized at 1/10 sample dilution, mean ± SEM, *n* = 6, and nonspecific binding in the absence of Env (*open triangles*, *n* = 1). *Open circles* show, placed arbitrarily on the x-axis, background binding to Env treated with 1/10 HIV− serum samples (*n* = 3). **(B)** *Right* axis, viral load with *dotted lines* indicating the assay detection limits at 400 and subsequently 50 viral copies per milliliter. **(C)** *Right* axis, p24-stimulated T cell proliferation and **(D)** IFNγ ELISPOTs/10^6^ PBMCs stimulated with gag, pol, env, and nef peptides. Dimeric rsFcγR activities at indicated times were compared using an unpaired Mann–Whitney *t*-test, **p ≤ .01.

Subjects SC24 and SC84 had undetectable VL on ART at recruitment before several short periods of STIs and a final STI of about 10 months. SC24 had two short STIs, and during the final extended STI, a spike in viremia (20,000 viral copies/mL at 46 months) was followed by a rapid increase in anti-Env IgG1 levels, dimeric rsFcγRIIa binding activity, and virological control. Virological control was not sustained, with VL fluctuating between 140 and 3,600 copies/mL in the following 9 months (Fig. 6A, B). The first STI for SC24 (42 months) had no detectable viral replication (<50 copies/mL) and low levels of both IgG and dimeric rsFcγRIIa binding activity (Fig. 6A). When assayed at low dilution, the initial STI trended toward an increase in both dimeric rsFcγRIIa and dimeric rsFcγRIIIa binding activities (Fig. 6B, C).

T cell response to changes in ART and viral replication in SC24 were also associated with possible long-term control of viremia after ceasing ART. The p24 proliferative response fluctuated during STIs and remained elevated immediately after the initial peak viral rebound during ART cessation (Fig. 6C). During the extended STI, the CD8 T cell response to env antigens increased ∼10-fold and remained elevated (Fig. 6D).

**FIG. 7.**
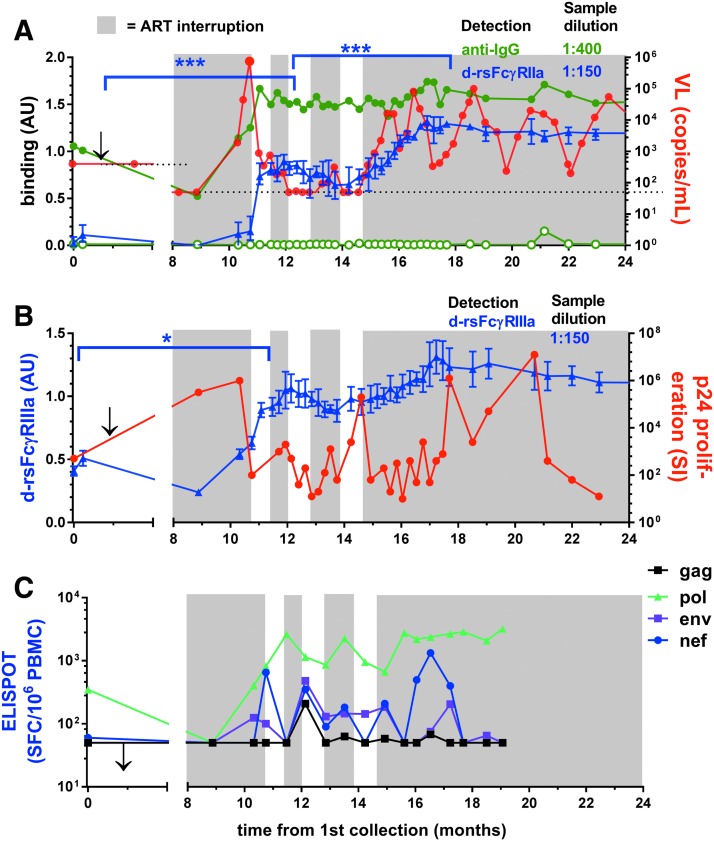
STI in patient SC84 stimulates anti-Env IgG with dimeric rsFcγR binding activity, and sustained ART interruption is marked by episodic control of viremia. **(A, B)** Dimeric rsFcγR binding to Env opsonized at 1/150 sample dilution, mean ± range, *n* = 3. **(A)** *Left* axis, dimeric rsFcγRIIa-H131 activity, normalized AU, and anti-IgG1 binding to a 1/400 dilution of samples with and without gp140 (*green closed* and *open* symbols, respectively); *right* axis, viral load with *dotted lines* indicating the assay detection limits at 400 and subsequently 50 viral copies per milliliter. The *arrow* marks the initiation of ART and periods of ART removal are shown with a *gray* background. **(B)** *Left* axis, dimeric rsFcγRIIIa activity, normalized AU, *right* axis p24-stimulated T cell proliferation and **(C)** IFNγ ELISPOTs/10^6^ PBMCs stimulated with gag, pol, env, and nef peptides. Activities at indicated times were compared using an unpaired Mann–Whitney *t*-test, *p < .05 and ***p < .001.

In contrast to SC24, the first STI for subject SC84 was longer and resulted in high viremia (>750,000 copies/mL) that was rapidly controlled by resumption of ART (Fig. 7A). This viremia stimulated marked increases in Ab levels and dimeric rsFcγRIIa and dimeric rsFcγRIIIa binding activities (Fig. 7A, B). During two short subsequent STI cycles, viral rebounds were greatly reduced (<750 copies/mL). After ceasing ART, further enhanced dimeric rsFcγR activities were contemporaneous with cyclic fluctuations in viremia with periodic control to <1,000 copies/mL.

The T cell proliferative response to p24 was sensitive to viral rebound, declining during peak viremia and then increasing during subsequent control when ART recommenced (Fig. 7B). This helper T cell response was boosted after ceasing ART with an inverse association with viremia during cyclic control in the absence of ART. The CD8 T cell responses increased proportionally with viral rebound during ART interruption and after ceasing ART (Fig. 7C). The p24 proliferative response eventually declined, whereas humoral responses were maintained upon 12-month follow-up.

## Discussion

This study of subjects treated with ART early after infection was designed to determine if short periods of viral antigen exposure experienced during STI could boost functional immune responses associated with viral containment, which are otherwise suppressed during continuous ART. With the aid of very frequent patient blood sampling during these STI cycles, we demonstrated boosting of both IgG responses, dimeric rsFcγR binding activity and T cell immunity, which contributed to virological control in some individuals. The study also confirmed that rapid decline in anti-envelope Ab titer occurs early during suppressive ART, but boosting of anti-Env Abs and FcγR binding activities was achieved after only a few STI cycles, even when viral rebound was low (e.g., 460 copies/mL). Boosted dimeric FcγR binding is indicative of increased IgG functionally during these STIs as these correlate with phagocytosis and natural killer (NK) cell ADCC activities.^31–35,37,44^

Therapeutic attempts to reactivate proviruses to clear viral reservoirs may benefit from non-neutralizing Abs with effector function against Env-expressing cells. While early initiation of ART is the essential standard care for HIV infection, it diminishes humoral and FcγR functional responses. Although only partial virological control was achieved in these pilot studies (i.e., SC24 and SC84), the capacity for a small number of short STIs with low viral recrudescence (i.e., SC21) to rapidly boost Ab levels and dimeric rsFcγR binding (e.g., SC21, SC24, and SC84) can help to guide therapeutic approaches. Taken together, our data support the concept of viral antigen vaccination during ART as part of an HIV eradication strategy.

In addition to boosted Ab responses, it is notable that T cell responses were maintained after cessation of ART, and prolonged proliferative responses may suggest provision of T cell help for CTL and humoral control of viremia. However, Env-specific humoral responses proved more durable than helper T cell responses after ART was discontinued. We therefore propose that dimeric FcγR binding could serve as a primary efficacy outcome measure in HIV-1 treatment and eradication strategies. A follow-up PBMC sample was not available to determine the long-term T cell response beyond 12 months without ART, but a plasma specimen taken 1 year after the study from SC84 demonstrated that humoral responses endured. A study of extended ART interruptions in chronically infected patients found boosted antibody titer, but not neutralization activity, suggesting that enhancement of Fc-mediated function is the likely humoral outcome of antigen exposure during STI.^45^ Under the cover of ART, this could possibly be achieved by vaccination with newer recombinant forms of envelope proteins. It is encouraging that influenza A vaccination studies showed robust responses by ART patients.^31^

That a few short STI cycles provided some immune-based virological containment in this study could be attributed to high immunocompetence and early treatment in our participants. In ART-treated chronically infected subjects with elevated CD4 counts, four consecutive short (2 weeks) ART interruptions stimulated small increases in antibodies to gp120 and more substantial increases were observed only after a fifth extended ART interruption.^45^ Our former study of panobinostat trial and SMART subjects found that longer ART interruption periods (>3 weeks, ∼2 months) with viral recrudescence to an estimated ∼300 copies/mL were required to stimulate increases in dimeric rsFcγRIIIa binding. NK-mediated ADCC was significantly boosted only after 12 consecutive months of STI.^46^ In this current study, short STI intervals and low viremia (460 copies/mL in SC21) in some instances rapidly (<2 weeks) boosted dimeric rsFcγR-binding antibodies. Other studies described high T cell-based immunocompetence in some patients treated early after infection in the T cell compartment,^43^ and in one case, partial virological control was associated with NAb raised during ART interruption.^47^

The dimeric rsFcγR assays used in this study recapitulate the intrinsic binding properties of cellular receptors combined with a requirement for two antibodies to present their Fc regions appropriately to be bridged by the receptor dimer.^5^ This was exemplified using near-saturating levels of three mAbs, which effectively opsonized gp140, but did not support dimeric rsFcγRIIa binding. Unlike the IgG titer, the dimeric rsFcγR assay is a unique measure that reflects two aspects of the functionality of the IgG response, namely the ability to bind each FcγR and the extent of opsonization by pairs of closely spaced Abs. Such opsonization depends on multiple Ab specificities (≥2) to appropriately spaced epitopes to be present in pAbs. Low dilution of samples allows high occupancy of gp140 to maximize formation of such IgG pairs. The slower loss of such dimeric rsFcγR binding compared with the anti-HIV IgG1 titer during ART indicates retention of Ab specificities in this diminishing titer. This polyclonality suggests that boosting of an existing diverse Ab response by vaccination under ART should be possible.

In two subjects with low initial anti-Env IgG1 titers (SC49 and SC74), the dimeric rsFcγRIIIa binding activity was greater compared with dimeric rsFcγRIIa. This indicates a qualitative difference in these early antibody responses. Favored dimeric rsFcγRIIIa binding activity may be due, in part, to the higher affinity of this receptor for IgG or specific glycosylation of the Fc region of IgG in the Ab response. The absence of a bisecting fucose in the Asn279-linked carbohydrate strongly increases Fc interaction with FcγRIIIa and ADCC activities.^48–51^ Although levels of IgG3 were very low, the relative level of opsonizing anti-Env IgG3 to competing IgG1 was higher in these subjects. IgG3 opsonization of model immune complexes showed high dimeric rsFcγR binding,^37^ which possibly reflects the high effector functionality of this IgG subclass.^52^ While this pilot STI study does not allow further conclusions, this remains notable given the correlation of anti-Env of the IgG3 subclass with protection in the RV144 trial^19^ and the IgG3 skewed anti-Env response in a subset of HIV controllers.^53^

This analysis of early ART-treated subjects found that while the Ab titer rapidly diminishes, a few short STIs were sufficient to boost immunity, including a marker of FcγR function. Boosting FcγR function by Env protein vaccination during ART may be built into HIV-1 treatment and eradication strategies, and for this component, the dimeric FcγR assay could define a standard, primary efficacy outcome.
